# Analysis of MLKL, RIP1 and RIP3 Immunostaining Markers in Human Liver Tissue from Fatal Yellow Fever Cases: Insights into Necroptosis

**DOI:** 10.3390/v17010003

**Published:** 2024-12-24

**Authors:** Vanessa do Socorro Cabral Miranda, Luiz Fabio Magno Falcão, Hellen Thais Fuzii, Marcos Luiz Gaia Carvalho, Jeferson da Costa Lopes, Arnaldo Jorge Martins Filho, Ana Cecilia Ribeiro Cruz, Raimunda do Socorro da Silva Azevedo, Jorge Rodrigues de Sousa, Mayumi Duarte Wakimoto, Pedro Fernando da Costa Vasconcelos, Juarez Antônio Simões Quaresma

**Affiliations:** 1 Departmento of Pathology, Evandro Chagas Institute, Ministry of Health, Ananindeua 67030-000, PA, Brazil; vanessacabralmiranda@gmail.com (V.d.S.C.M.); marcosgaia@outlook.com (M.L.G.C.); jefersonchaz@hotmail.com (J.d.C.L.); arnaldofilho@iec.gov.br (A.J.M.F.); anacecilia@iec.gov.br (A.C.R.C.); raimundaazevedo@iec.gov.br (R.d.S.d.S.A.); krekrodrigues@gmail.com (J.R.d.S.); pedrovasconcelos@iec.gov.br (P.F.d.C.V.); 2 Departmento of Pathology, State University of Para, Belem 66050-540, PA, Brazil; fabiofalcao@uepa.br; 3 Tropical Medicine Center, Federal University of Para, Belem 66055-240, PA, Brazil; hellenfuzii@gmail.com; 4 Evandro Chagas National Institute of Infectious Diseases (INI-FIOCRUZ), Oswaldo Cruz Foundation, Rio de Janeiro 21040-360, RJ, Brazil; mayumi.wakimoto@ini.fiocruz.br; 5 Department of Infectious Disease, School of Medicine, Sao Paulo University, Sao Paulo 01246-930, SP, Brazil

**Keywords:** yellow fever, necroptosis, liver parenchyma

## Abstract

Necroptosis is a regulated form of cell death implicated in several pathological conditions, including viral infections. In this study, we investigated the expression and correlation of necroptosis markers MLKL, RIP1 and RIP3 in human liver tissue from fatal cases of yellow fever (YF) using immunohistochemistry (IHC). The liver samples were obtained from 21 YF-positive individuals and five flavivirus-negative controls with preserved liver parenchymal architecture. The cases underwent histopathological analysis, followed by tissue immunostaining with the immunohistochemical method of streptavidin–biotin peroxidase. Using the in situ method, we evaluated the centrilobular zone (Z3), midzonal zone (Z2), periportal zone and portal tract (PT) of human liver parenchyma with markers for necroptosis, RIPK1, RIPK3 and MLKL. A quantitative analysis revealed a significantly higher expression of MLKL, RIP1 and RIP3 in the liver parenchyma of YF cases compared to controls in different zones (Z3, Z2, Z1) and portal tracts (PTs) of the liver, especially in zone 2. Immunostaining confirmed the localization of MLKL, RIP1 and RIP3 in hepatocytes and inflammatory infiltrates, highlighting their involvement in the pathogenesis of YF. A Pearson correlation analysis demonstrated significant correlations among necroptosis markers, which indicates their coordinated regulation during YF-induced liver injury.

## 1. Introduction

Yellow fever (YF) is an arboviral disease transmitted by hematophagous mosquitoes that can present a spectrum of clinical manifestations ranging from asymptomatic cases to severe forms with hemorrhage. It is a disease transmitted by mosquitoes infected with the Yellow Fever Virus (YFV) belonging to the *Flavivirus* genus in Africa, and it is maintained in wild cycles by non-human primates (NHP) and hematophagous arthropods of the *Haemagogus* and *Sabethes* genera in South America. Its lethality ranges from 20% to 50% [1].

The urban cycle encompasses *Aedes aegypti* and human interactions, posing the most significant public health concern. The resurgence of this urban cycle in the Americas, coupled with vulnerable human populations, has raised alarms among public health authorities. This is primarily due to inadequate vaccination coverage in both non-endemic and endemic regions [1,2,3,4]. After the virus enters the human body through the bite of the transmitting mosquito, it quickly reaches the lymph nodes and spreads into the bloodstream. Viral replication begins in the lymph nodes. After the release of virions into the bloodstream, a period known as viremia, organs such as the liver, kidneys and spleen are also infected [5,6,7,8].

From the point of view of the pathophysiology of yellow fever, with the triggering of immune evasion strategies adopted by the virus, liver failure becomes a central problem where, in the phase of intense viral replication, the exacerbated immune response of the host leads to the massive propagation of the activation of cell-death mechanisms that culminate in severe hepatocyte necrosis. As a consequence, jaundice, hepatomegaly, the elevation of transaminases, the presence of hemorrhage, hypovolemic shock and renal dysfunction are factors that contribute significantly to acute liver failure in fatal cases. Apoptosis and necrosis have long been considered the main processes of cell death in YF infection, but it is believed that necroptosis may play an important role in the cell death of hepatocytes [9,10,11]. Necroptosis, a recently recognized type of programmed cell death, contributes to inflammation and various diseases such as cancer, stroke and kidney disease [12]. When triggered by various stimuli, necroptosis is initiated by the activation of receptor-interacting protein kinase 1 (RIP1). Activated RIP1 interacts with RIP3 through their RIP homotypic interaction motifs, phosphorylates RIP3 and forms an RIP1/RIP3 complex called the necrosome [13]. Then, mixed lineage kinase domain-like protein (MLKL) is recruited and phosphorylated by RIP3 in the necrosome [14]. Phosphorylated MLKL monomers aggregate to form oligomers and translocate to the plasma membrane to execute necroptosis [14,15].

Although multiple cell death pathways are involved in YF, including apoptosis and necrosis, the effect of necroptosis remains largely unknown [2,11,16,17,18,19]. In this study, our aim was to explore the activation of necroptosis in YF and identify the role of RIP1/RIP3/MLKL-mediated necroptosis in human liver tissue.

## 2. Materials and Methods

### 2.1. Patients, Samples and Diagnosis of Yellow Fever Infection

This study used 26 human liver biopsies. Among them, 21 samples of fatal cases of YF were confirmed through obtaining positive results for the virus by reverse transcriptase reaction followed by polymerase chain reaction (RT-PCR) and immunohistochemistry (one or both methods). In addition, five control samples from patients with preserved liver architecture tested negative for YF and other flaviviruses circulating in Brazil, according to the death verification service (the Renato Chaves Scientific Expertise Center) in the city of Belém in the state of Pará, Brazil. The confirmation of the diagnosis for the positive cases of YF was based on the studies of Melo et al. [8] and Olimpio et al. [16], including histopathological, immunohistochemical and real-time quantitative RT-PCR (RT-qPCR) analyses. For RT-qPCR, samples were processed using the 7500 Fast Real-Time PCR System (Applied Biosystems, Waltham, MA, USA) and the AriaMx Real-Time PCR System (Agilent Technologies, Santa Clara, CA, USA) using two RT-qPCR kits: (1) the Superscript III^®^ Platinum^®^ One-Step Quantitative RT-PCR System (Invitrogen, Waltham, MA, USA) and (2) the QuantiTect^®^ Probe RT-PCR (Qiagen, Germantown, MD, USA). For the histopathological diagnosis, the paraffin-embedded biopsies were cut into 5 μm sections and stained with the hematoxylin–eosin method. Table 1 shows detailed information about the patients included in this study.

### 2.2. Ethics Statement

The patient samples were obtained and processed as part of the response measures to the surveillance of the YFV epidemic in Brazil on an emergency basis, as defined by the Ministry of Health. This study was approved (No. 2.824.592) by the Research Ethics Committee (CEP) of the Evandro Instituto Chagas (IEC). All of the methods were performed in accordance with the relevant guidelines and regulations approved by the CEP/IEC and the Brazilian Ministry of Health rules and regulations for studies with biological samples.

### 2.3. Immunohistochemistry (IHC)

Immunostaining of the hepatic tissues with antibodies specific for MLKL (Abnova, Taiwan, TW, H00197259-M02, dilution 1:50), RIP1 (Abcam, Cambridge, UK, ab72139, dilution 1:50) and RIP3 (Abcam, Cambridge, UK, ab152130, dilution 1:50) was performed using the streptavidin–biotin peroxidase immunohistochemical method (SABC) [20] and adapted according to Olímpio et al. [16]. Briefly, the tissue samples were deparaffinized in xylene and hydrated in a decreasing series of ethanol (90%, 80%, 70%). The liver sections were incubated in 3% hydrogen peroxide for 45 min to block endogenous peroxidase. Incubation in citrate buffer, pH 6.0, for 20 min at 90 °C was realized to recover antigens. Non-specific proteins were blocked by incubating the sections in 10% skim milk for 30 min. The histological sections were then incubated overnight with the primary antibodies diluted in 1% bovine serum albumin. The slides were immersed in 1 × PBS and incubated with the biotinylated secondary antibody (LSAB; DakoCytomation, Glostrup, Denmark) in an oven for 30 min at 37 °C. The slides were immersed in 1 × PBS and incubated with streptavidin peroxidase (LSAB; DakoCytomation) for 30 min at 37 °C. For visualization, the specimens were treated with a chromogenic solution (0.03% diaminobenzidine and 3% hydrogen peroxide). Finally, the histological sections were washed in distilled water, counterstained with Harris hematoxylin for 1 min, dehydrated in ethanol (70%, 80%, 90%) and deparaffinized in xylene.

### 2.4. Quantitative Analysis and Photo-Documentation

The markers that characterize in situ necroptosis were analyzed with the Axio Imager Z1 microscope, where the expression of each marker was quantified from the random selection of 10 fields, each divided into areas smaller than 10 × 10 μm, delimited by a 0.0625 mm^2^ grid. The microscopic scanning for each field was established in the hepatic acinus, including the zones (Z3: pericentral zone; Z2: midzonal zone; Z1: periportal zone; PT: portal tract) that comprise the Rappaport space. These zones were analyzed for both the fatal cases that were positive for yellow fever and for those in the control group.

### 2.5. Statistical Analysis

The data were stored in a Microsoft Excel 2016 spreadsheet and analyzed using GraphPadPrism 9.0. The numerical variables were expressed as the mean, median, standard deviation and variance. A one-way ANOVA, Tukey’s test and the Pearson correlation were also applied; the results were considered statistically significant at *p* < 0.05.

## 3. Results

### Expression of RIP1, RIP3 and MLKL in the Hepatic Parenchyma in Fatal Yellow Fever Cases

**Figure 1:**
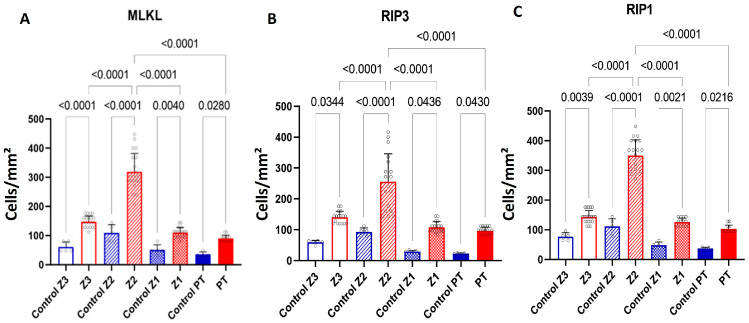
Quantitative analysis of MLKL (**A**), RIP3 (**B**) and RIP1 (**C**) in zones Z3, Z2, Z1 and PT in the liver parenchyma of fatal cases of human YF compared to negative control.

**Figure 2:**
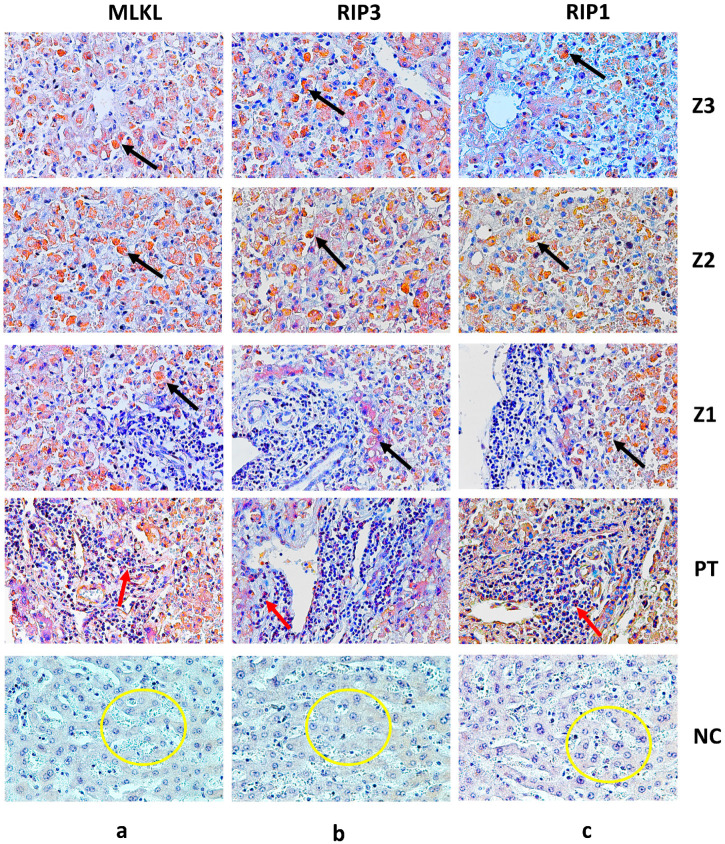
Immunostaining for MLKL (Z3-a, Z2-a, Z1-a), RIP3 (Z3-b, Z2-b, Z1-b) and RIP1 (Z3-c, Z2-c, Z1-c) in hepatocytes (black arrow) and PT-a, PT-b and PT-c in inflammatory infiltrates (red arrow). Preservation of hepatic parenchyma in control cases and minimal expression of MLKL (**a**), RIP1 (**b**) and RIP3 (**c**) (yellow circle). Magnification of 400× (scale: 20 μm). Z3: Centrilobular zone; Z2: midzonal zone; Z1: periportal zone; PT: portal tract; NC: normal control.

The results obtained from the analyses of the fatal cases that were positive for YFV compared with the control samples revealed significant differences of RIP1, RIP3 and MLKL, with highly significant *p* values, as observed in Table 2 and Figure 1. Furthermore, the immunostaining for each marker demonstrated that, in the zones (Z3, Z2 and Z1), the expression of the markers was predominantly found in hepatocytes (Figure 2), while in the portal tract, it was centralized in the inflammatory infiltrates composed mainly of lymphomononuclear cells (Figure 2).

## 4. Discussion

The current study provides crucial insights into the expression and correlation of necroptosis markers (MLKL, RIP1 and RIP3) in liver tissue from fatal yellow fever (YF) cases, shedding light on the underlying mechanisms of YF pathology. The immunostaining results confirmed the presence of MLKL, RIP1 and RIP3 in hepatocytes and inflammatory infiltrates (Table 2, Figure 1 and Figure 2). The preservation of the hepatic parenchyma and the minimal expression of these markers in the control cases further support their specific involvement in YF pathology. The quantitative analysis revealed a significant upregulation of these markers in YF cases, particularly in the midzonal zone, followed by the pericentral and periportal zones and the inflammatory infiltrates in smaller expressions (Table 2, Figure 1 and Figure 2). This spatial variation suggests the involvement of necroptosis in liver damage and hepatocyte loss as having distinct roles in necroptosis induction and execution during YF infection.

**Figure 3:**
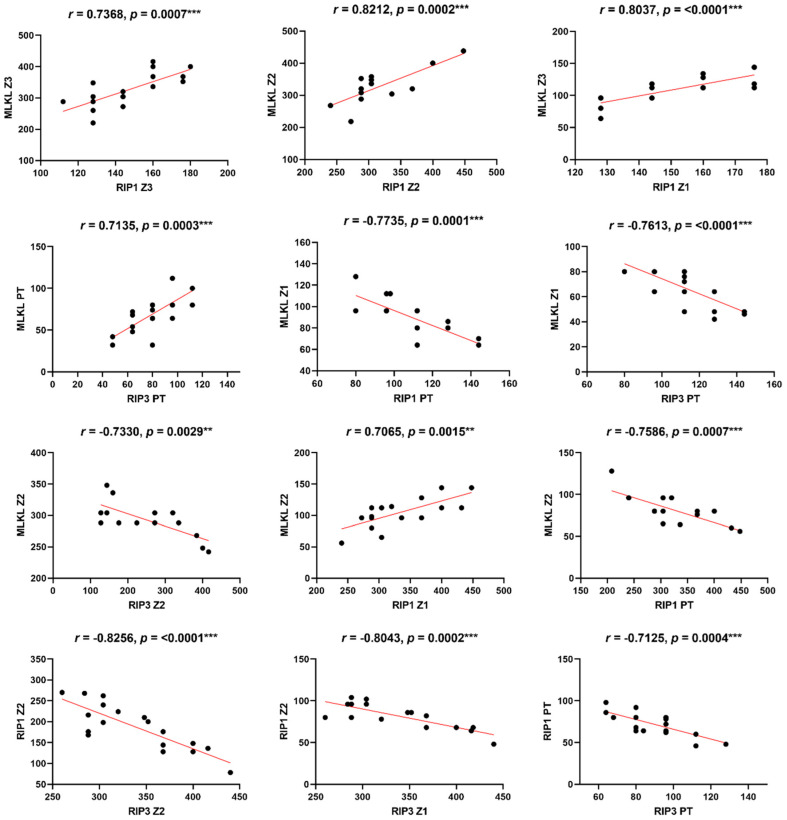
Linear correlations among receptors that characterize in situ necroptosis response in fatal cases of YFV. Pearson correlation: ** *p* < 0.001; *** *p* < 0.0001.

The Pearson correlation analysis revealed significant correlations among MLKL, RIP1 and RIP3 in YF cases (Table 3, Figure 3). The positive correlations between MLKL and RIP1, as well as between MLKL and RIP3, indicate their cooperative role in necroptosis signaling. Moreover, the negative correlations observed among MLKL, RIP1 and RIP3 in certain liver zones and portal tracts imply the existence of complex regulatory mechanisms that vary across different tissue microenvironments (Table 3, Figure 3). Such scenarios indicate the dichotomy of responses of the association of markers, which can influence the cellular response to tissue damage that can contribute to the deterioration of liver tissue as well as aggravate inflammation and liver injury. This can cause a vicious cycle in which the virus, through immune evasion strategies, can modulate cellular injury in different aspects that impact cell swelling and membrane rupture, which is a hallmark of necroptosis. Supporting these findings, Vandenabeele et al. [21] demonstrated the crucial role of RIP1 and RIP3 in necroptosis signaling pathways. The study identified RIP1 and RIP3 as key players in the formation of the necrosome complex, which leads to the execution of necroptosis. In our study, we observed significantly higher expression levels of RIP1 and RIP3 markers in liver tissue from fatal YF cases compared to controls. This finding supports the involvement of necroptosis in YF pathogenesis, suggesting that RIP1 and RIP3 may contribute to hepatocellular necroptosis and tissue damage.

Furthermore, Wu et al. [15] highlighted the significance of MLKL in mediating necroptosis-associated inflammation. MLKL is a downstream effector molecule activated by RIP3, leading to plasma membrane disruption and the release of intracellular contents, triggering inflammation. During necroptosis, the formation of RIPK1/3 heterodimers gives rise to a complex that triggers the NF-kB-mediated pro-inflammatory response, facilitating the release of cytokines/chemokines [22,23,24]. Additionally, the secretion of damage-associated molecular patterns (DAMPs), such as ATP and HMGB1, is notably heightened during necroptosis [25,26]. As a consequence of these signaling cascades, morphological changes distinct from apoptosis become evident in necroptosis, including organelle swelling and cellular lysis [27]. This specific morphology is discernible in Figure 2 (black arrows), indicating the occurrence of necroptosis in a severe yellow fever infection. This is further supported by elevated levels of MLKL expression in the hepatic tissue of fatal yellow fever cases (Table 2, Figure 1) as well as by the positive correlation between MLKL and RIP1/3 expression levels (Table 3, Figure 3).

Review work by Verburg et al. [28] discussed the contribution of RIP3 and MLKL to immunopathology in viral hepatitis. The study demonstrated that the activation of RIP3 and MLKL promotes liver inflammation and tissue damage. Our results are consistent with these findings, as we observed upregulated expression levels of RIP1, RIP3 and MLKL in liver tissue from fatal YF cases. The positive correlations observed among these markers suggest their involvement in promoting hepatocellular necroptosis and inflammation in YF.

While our study focused on YF, this research provides insights into the crosstalk between different forms of regulated cell death. Necroptosis and pyroptosis share common signaling pathways and may contribute to tissue damage and inflammation [29]. Although our study did not investigate pyroptosis specifically, the findings highlight the complexity of cell death mechanisms in viral infections and their potential impact on disease progression.

Overall, the literature supports our results, indicating the involvement of necroptosis and the upregulation of MLKL, RIP1 and RIP3 markers in liver tissue from fatal YF cases. The positive correlations observed among these markers further reinforce their interplay and potential contribution to hepatocellular necroptosis and tissue damage. Understanding the molecular mechanisms of necroptosis and its association with YF pathogenesis may pave the way for the development of targeted therapies to mitigate liver injury and improve patient outcomes in YF cases.

## 5. Conclusions

Our study provides quantitative evidence for the upregulation of the necroptosis markers MLKL, RIP1 and RIP3 in human liver tissue from fatal YFV cases. The differential expression patterns of these markers in different liver zones and portal tracts suggest a spatially regulated involvement of necroptosis in the pathogenesis of yellow fever. Furthermore, the observed correlations among MLKL, RIP1 and RIP3 expression levels in the YFV cases support their cooperative role in necroptosis induction during YFV infection. Further research is needed to elucidate the underlying molecular mechanisms and the potential therapeutic implications of targeting necroptosis in yellow fever.

## Figures and Tables

**Table 1:** Characterization of patients with YF according to gender, age, state, year and duration of disease.

Case	Patient	Gender	Age	State	Year	IT *
1	001/00	M	25	Tocantins	2000	8
2	106/00	M	75	Goiás	2000	NR **
3	108/00	M	49	Goiás	2000	7
4	494/00	M	NR **	Distrito Federal	2000	NR **
5	251/00	M	16	Mato Grosso do Sul	2000	6
6	252/00	M	49	Goiás	2000	NR **
7	253/00	M	23	Goiás	2000	NR **
8	255/00	M	NR **	Goiás	2000	NR **
9	291/00	M	NR **	Goiás	2000	NR **
10	158/00	M	33	Goiás	2000	NR **
11	063/03	M	NR **	Minas Gerais	2003	NR **
12	339/04	M	36	Amazonas	2004	11
13	019/08	M	64	Goiás	2008	7
14	273/08	M	57	Goiás	2008	7
15	068/08	F	65	Goiás	2008	2
16	095/08	M	42	Goiás	2008	3
17	143/08	M	37	Distrito Federal	2008	NR **
18	361/15	F	53	Rio Grande do Norte	2015	4
19	062/16	M	35	Goiás	2016	NR **
20	346/16	M	15	Goiás	2016	7
21	369/16	M	27	Goiás	2016	1

* IT = Illness time/** NR = No registry.

**Table 2:** Quantitative analysis of necroptosis markers in hepatic parenchyma in fatal YFV cases compared to control cases. Z3: Pericentral zone; Z2: midzonal zone; Z1: periportal zone; PT: portal tract. One-way ANOVA *** *p* < 0.0001; Tukey *** *p* < 0.0001; ** *p* < 0.001; * *p* < 0.05.

Markers	Z3 (Cells/mm^2^)	Z2 (Cells/mm^2^)	Z1 (Cells/mm^2^)	PT (Cells/mm^2^)	ANOVA (*p* ≤ 0.05)
RIP1 Control	143.20 ± 21.75 76.80 ± 13.39	350.40 ± 52.40 112.00 ± 25.30	125.70 ± 13.83 48.40 ± 9.94	102.4 ± 11.19 36.80 ± 4.60	***
Tukey (*p* ≤ 0.05)	***	***	**	*	
RIP3 Control	140.2 ± 19.41 59.20 ± 6.57	255.10 ± 90.92 92.80 ± 13.39	107.3 ± 18.87 30.20 ± 4.38	96.33 ± 12.06 22.20 ± 2.68	***
Tukey (*p* ≤ 0.05)	***	***	*	*	
MLKL Control	147.80 ± 19.53 60.80 ± 17.53	318.50 ± 63.38 108.80 ± 28.62	109.70 ± 17.73 51.20 ± 17.53	89.60 ± 11.79 36.00 ± 8.00	***
Tukey (*p* ≤ 0.05)	***	***	**	*	

**Table 3:** Linear correlations among markers of necroptosis in fatal yellow fever cases of moderate to strong correlation. Pearson correlation: *** *p* < 0.0001; ** *p* < 0.001; * *p* < 0.05.

Correlation	r	*p*-Value
MLKL Z3 x RIP1 Z3	0.5804	0.0058 **
**MLKL Z3 x RIP1 Z2**	**0.7368**	0.0007 ***
**MLKL Z3 x RIP1 Z1**	**0.8037**	<0.0001 ***
**MLKL Z2 x RIP1 Z2**	**0.8212**	0.0002 ***
**MLKL Z2 x RIP1 Z1**	**0.7065**	0.0015 **
**MLKL Z2 x RIP1 PT**	**−0.7586**	0.0007 ***
**MLKL Z1 x RIP1 PT**	**−0.7735**	0.0001 ***
MLKL Z3 x RIP3 Z3	0.4726	0.0305 *
MLKL Z3 x RIP3 Z2	0.6764	0.0079 **
MLKL Z3 x RIP Z1	−0.4788	0.0381 *
**RIP3 Z2 x MLKL Z2**	**−0.7330**	0.0029 *
**MLKL Z1 x RIP3 PT**	**−0.7613**	<0.0001 ***
**MLKL PT x RIP 3 PT**	**0.7135**	0.0003 ***
RIP1 Z3 x RIP3 PT	−0.5957	0.0044 **
**RIP1 Z2 x RIP3 Z2**	**−0.8256**	<0.0001 ***
**RIP1 Z2 x RIP3 Z1**	**−0.8043**	0.0002 ***
RIP1 Z2 x RIP3 PT	−0.6483	0.0015 **
RIP1 Z1 x RIP3 Z1	−0.5047	0.0327 *
**RIP1 PT x RIP3 PT**	**−0.7125**	0.0004 ***
RIP1 Z3 x RIP3 Z2	0.4601	0.0475 *

## Data Availability

The raw data supporting the conclusions of this article will be made available by the authors without undue reservation.
